# An RNA sequencing transcriptome analysis of the high-temperature stressed tall fescue reveals novel insights into plant thermotolerance

**DOI:** 10.1186/1471-2164-15-1147

**Published:** 2014-12-19

**Authors:** Tao Hu, Xiaoyan Sun, Xunzhong Zhang, Eviatar Nevo, Jinmin Fu

**Affiliations:** Key Laboratory of Plant Germplasm Enhancement and Specialty Agriculture, Wuhan Botanical Garden, Chinese Academy of Science, Wuhan, 430074 Hubei P.R. China; Department of Crop and Soil Environmental Sciences, Virginia Polytechnic Institute and State University, Blacksburg, VA 24061 USA; Institute of Evolution and the International Graduate Center of Evolution, University of Haifa, Haifa, 31905 Israel

**Keywords:** High-temperature stress, RNA sequencing, Cell cycle, Energy metabolism, Tall fescue

## Abstract

**Background:**

Tall fescue (*Festuca arundinacea* Schreb.) is major cool-season forage and turf grass species worldwide, but high-temperature is a major environmental stress that dramatically threaten forage production and turf management of tall fescue. However, very little is known about the whole-genome molecular mechanisms contributing to thermotolerance. The objectives of this study were to analyzed genome-wide gene expression profiles in the leaves of two tall fescue genotypes, heat tolerant ‘PI578718’ and heat sensitive ‘PI234881’ using high-throughput RNA sequencing.

**Results:**

A total of 262 million high-quality paired-end reads were generated and assembled into 31,803 unigenes with an average length of 1,840 bp. Of these, 12,974 unigenes showed different expression patterns in response to heat stress and were categorized into 49 Gene Ontology functional subcategories. In addition, the variance of enrichment degree in each functional subcategory between PI578718 and PI234881 increased with increasing treatment time. Cell division and cell cycle genes showed a massive increase in transcript abundance in heat-stressed plants and more activated genes were detected in PI 578718 by Kyoto Encyclopedia of Genes and Genomes pathways analysis. Low molecular weight heat shock protein (LMW-HSP, HSP20) showed activated in two stressed genotypes and high molecular weight HSP (HMW-HSP, HSP90) just in PI578718. Assimilation such as photosynthesis, carbon fixation, CH_4_, N, S metabolism decreased along with increased dissimilation such as oxidative phosphorylation.

**Conclusions:**

The assembled transcriptome of tall fescue could serve as a global description of expressed genes and provide more molecular resources for future functional characterization analysis of genomics in cool-season turfgrass in response to high-temperature. Increased cell division, LMW/HMW-HSP, dissimilation and antioxidant transcript amounts in tall fescue were correlated with successful resistance to high temperature stress.

**Electronic supplementary material:**

The online version of this article (doi:10.1186/1471-2164-15-1147) contains supplementary material, which is available to authorized users.

## Background

High-temperature stress has devastating effects on plant growth and productivity, and is a growing concern in the context of global climate change [1, 2]. Temperatures 5°C above optimal growing conditions will induce heat shock or heat stress to plants [3]. Every year, there is substantial crop yield loss due to the elevated temperature and drought stress [4]. The development of genetically heat-resistant plants has been considered as a promising approach for alleviating the threats from high-temperature stress [5].

Heat tolerance of plants is a complex multigenic process with different regulatory mechanisms sets throughout the gene network during plant development [6, 7]. Larkindale *et al*. [8, 9] reported that each of 45 *Arabidopsis* mutants had different responses to high temperature, which were involved in signaling pathways [abscisic acid (ABA), ethylene (ET), salicylic acid (SA), calcium, oxidative burst] and reactive oxygen metabolism (ascorbic acid or glutathione production, catalase). Additionally, ABA, SA or active oxygen molecule may serve as one signal to regulate gene chain in heat tolerant *Arabidopsis*. Although many researches on heat-stress tolerance have advanced considerably in recent years, genome-wide comparisons in plants have not yet been made using the next-generation high-throughput sequencing technologies. Determining the multigenic net regulatory mechanisms of plant response to high temperature, particularly at the transcriptomic level, will be helpful in the development of heat-tolerant species.

Tall fescue (*Festuca arundinacea* Schreb.) is a forage and cool-season turfgrass species grown widely in the temperate regions of the world such as in United States, China, Japan, Australia and many countries in Europe because of its agronomic importance [10–12]. However, high temperature (30–41°C) is one of the limiting factors affecting forage production and turf management of tall fescue in the south such as the Great Plains of the U.S. and south of the Yangtze Rive in China [3].

The genome size of tall fescue is approximately 6 × 10^3^ Mbp and about 14 times larger than that of rice (*Oryza sativa* L.) [13]. To date, complete genome sequences of tall fescue are not achieved. Although 59, 578 expressed sequence tags (ESTs) had been deposited in the NCBI GenBank database as of June 2014, molecular resouces of tall fescue are still limited because of the lack of genomic and transcriptomic information. An accelerated effort to acquire transcriptomes of tall fescue in response to high-temperature stress will be helpful in the development of heat-tolerant tall fescue cultivars. In the current study, two tall fescue genotypes, heat tolerant PI578718 and heat sensitive PI234881 identified from 120 tall fescue genotypes in our previous study (Additional file 1) [14], were used to investigate responses to high temperature at a global transcriptional level using an high-throughput RNA sequencing (RNA-Seq) approach. The objectives of this study were (1) to provide an initial investigation of genome-wide transcriptomic analysis; (2) to reveal novel insights into the regulation pattern of certain candidate genes in the critical metabolic pathways response to heat stress in cool-season turfgrass species.

## Results and discussion

### Analyses of RNA-Seq data

Cool-season turfgrass and forage grass species are especially sensitive to heat stress [1, 14]. Heat stress-induced injury may take place on the leaf at first [3]. Leaves of heat tolerant PI578718 and heat sensitive PI234881 were collected at 12 and 36 hour after treatment (HAT; the time point of the richest gene expression) based upon earlier studies [3, 15] and our pre-experiment for RNA-Seq analysis. The results of this study described the gene-level transcriptome in response to heat stress conditions in two tall fescue genotypes differing in high-temperature tolerance. Using the Illumina HiSeq 2000 system (Illumina Inc. San Diego, CA), a total of 262 million high-quality 100-bp paired-end reads were sequenced and assembled into a reference transcriptome of tall fescue (Table 1), which is also the first transcriptome library in the grass species to be reported. A total of 241,537 contigs were produced with a maximum length of 17,976 bp, average length of 596.18 bp and N50 of 821 bp for PI578718; a total of 253,100 contigs with a maximum length of 17,633 bp, average length of 590.61 bp and N50 of 806 bp for PI234881 were assembled, respectively. PI578718 obtained 484,390 transcripts with a maximum length of 15,993 bp, average length of 1,218 bp and N50 of 1,952 bp; PI234881 obtained 483,175 transcripts with a maximum length of 17,029 bp, average length of 1,192 bp and N50 of 1,950 bp.

**Table 1:** **Overview of the obtained RNA-Seq data from the Trinity** ***de novo*** **assembly program in tall fescue leaves induced by heat stress**

Assembly statistic (Contig)	Accession name	Total Length (bp)	Sequence No.	Max Length (bp)	Ave Length (bp)	N50	>N50 Reads No.	GC%	
	PI 578718	143,998,502	241,537	17,976	596.18	821	42,413	47.36	
	PI 234881	149,482,961	253,100	17,633	590.61	806	44,521	47.41	
**Assembly statistic (Transcript)**		**Total Length (bp)**	**Locus No.**	**Max Length (bp)**	**Ave Length (bp)**	**N50**	**>N50 Reads No.**	**GC%**	**Transcript No.**
	PI 578718	589,844,441	136,887	15,993	1,218	1,952	99,013	47.04	484,390
	PI 234881	575,842,899	150,053	17,029	1,192	1,950	94,941	47.15	483,175
**Unigene**		**Total Length (bp)**	**Unigene No.**	**Max Length (bp)**	**Ave Length (bp)**	**N50**	**>N50 Reads No.**	**GC%**	
		58,517,851	31,803	17,029	1,840	2,584	7,682	49.28	

Using the Trinity software, a final total of 31,803 unigenes (N50 of 2584 bp) from 967,565 transcripts were generated, whose average length of 1,840 bp was longer than those reported in other plants, such as litchi (*Litchi chinensis* Sonn.; 601 bp) [16], bracken fern (*Pteridium aquilinum*; 547.2 bp) [17], tea (*Camellia sinensis*; 355 bp) [18]. 15,689 (49.33%) unigenes of these 31,803 unigenes were matched with the homologs in databases with known function for tall fescue. A transcriptome atlas of two wheat genotypes response to heat treatments using microarray analyses only detected the expression of 22,720 probe sets [4]. Other suppression subtractive hybridization (SSH)-based studies involved in high temperature response just detected the expression of 1,090 transcripts in fescue (*Festuca* sp.) seeds of PI 297901 and PI283316 [3]. The unigenes obtained from a recent whole-genome-wide transcriptional analysis in two tall fescue genotypes under stress could greatly enrich the gene expression library response to high temperature in the grass family, especially in turfgrass and forage grasses. This Transcriptome Shotgun Assembly project has been deposited at DDBJ/EMBL/GenBank under the accession GBYN00000000. The version described in this paper is the first version, GBYN01000000.

### GO enrichment of the tall fescue heat transcriptome

**Figure 1:**
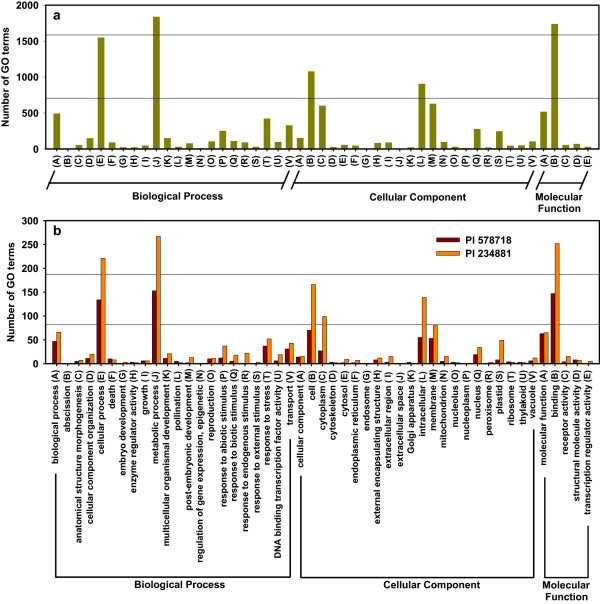
**Functional categorization of assembled unigenes based on Gene Ontology (GO) classification in leaves of two tall fescue genotypes (heat-tolerant PI 578718 and heat-sensitive PI 234881). (a)** functional categorization of entire genes for all treatment lines of two genotypes (green bars); **(b)** functional categorization of genes for 36 h heat-stressed PI 578718 and PI 234881. The unigenes were summarized in three main GO categories (biological process, cellular component and molecular function) and 49 subcategories. The y-axis indicates the number of unigenes in each class and the x-axis indicates the subcategories.

On the basis of Gene Ontology (GO) analysis, 12,974 unigenes were identified to be differential heat response between the two genotypes under different heat treatments, and were categorized into 49 GO functional subcategories (http://www.geneontology.org), which were summarized into three main categories: biological process (5,979; 46.08%), cellular components (4,583; 35.32%) and molecular function (2,412; 18.59%; Figure 1a). Genes encoding cellular process (1,549; 25.91%) and metabolic process (1,839; 30.76%) proteins were the most enriched in the biological process category. The result is in agreement with the result from litchi fruits in response to shading stress [16]. Genes encoding cellular process (1,549; 25.91%) and metabolic process (1,839; 30.76%) proteins were the most enriched in the biological process category. Within the cellular components, proteins related to cell (1,079; 23.54%), cytoplasm (602; 13.14%), intracellular (906; 19.77%) and membrane (629; 13.72%) were enriched. Under the molecular function category, the molecular function (518; 21.48%) and binding (1,738; 72.06%) were the most highly represented GO terms (Figure 1a).

The number of functional subcategories and variance degree in these three categories increased along with increased treatment time (Additional files 2, 3, 4). PI 578718, one of the most heat-hardy wild tall fescue genotype, may have a larger number of genes enriching in GO categories. However, RNA-Seq results did not support this hypothesis. On the contrary, a greater number of genes (1,568 vs. 1,363) were seen in the heat sensitive PI234881. In addition, the gene variance degree between the two genotypes in the same GO subcategories reached the maximum at the longest treatment time. At 36 HAT, genes involved in 18 subcategories from three main GO categories in the heat-sensitive genotype had more than two times enrichment than in the heat- tolerant genotype. Furthermore, genes, just encoding abscission, death, enzyme regulator activity, pollination, cytoskeleton, Golgi apparatus, nucleolus, ribosome, structural molecule activity and thylakoid proteins, were more enriched in the heat-tolerant genotype than in the heat-sensitive genotype at 36 HAT (Figure 1b). Similar results were reported in grapevine shoot apices in response to cold stress [19].

### COG functional annotation and classification of the tall fescue transcriptome

**Figure 2:**
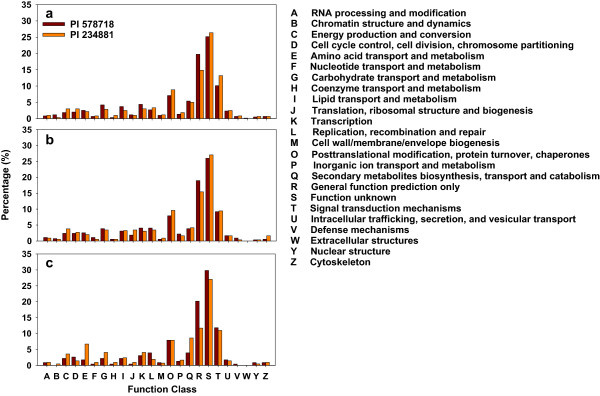
**Histogram of Clusters of evolutionary genealogy of genes: Non-supervised Orthologous Groups (eggNOG) classification.** All unigenes were aligned to the Clusters of Orthologous Groups (COG) and Eukaryotic Orthologous Group (KOG) database to predict and classify possible functions and separated into 24 clusters. **(a)**, **(b)** and **(c)** represent the eggNOG function classification from both tall fescue genotypes under heat stress for 0, 12 and 36 h, respectively.

The function of assembled unigenes was further evaluated using Clusters of Orthologous Groups (COG) analysis. Overall, 21,654 (68.09%) unigenes were assigned to 24 COG categories (Figure 2). In the 24 COG categories, the cluster for ‘function unknown’ (27.55%) represented the largest group, followed by ‘general function prediction only’ (17.96%), ‘signal transduction mechanisms’ (9.04%) and ‘posttranslational modification, protein turnover, chaperones’ (6.98%), ‘transcription’ (4.91%), ‘carbohydrate transport and metabolism’ (3.74%), ‘translation, ribosomal structure and biogenesis’ (3.03%), ‘secondary metabolites biosynthesis, transport and catabolism’ (2.90%) and ‘lipid transport and metabolism’ (2.90%). Only a small portion of the unigenes were assigned to ‘cell motility’ (0.046%) or ‘undetermined’ (0.005%). Compared with previous studies on peanut (*Arachis hypogaea*, 19,000, 26.20%) [20], longan (*Dimocarpus longan*, 17,118, 24.84%) [21], litchi (10,234, 30.07%) [16], these tall fescue unigenes could be more annotated through COG system (21,654, 68.09%). 27.55% unigenes in tall fescue were clustered in ‘function unknown’, because the COG functional annotation analysis was searched against five databases from close kinship species: Brachypodium hereafter (*Brachypodium distachyon*), wheat (*Triticum* spp.), rice (*Oryza sativa* Linn.), maize (*Zea mays* L.), sorghum [*Sorghum bicolor* (L.) Moench]. However, the other unigenes were relatively uniformly distributed in the COG categories, except the unigenes in ‘general function prediction only’ (17.96%) and ‘posttranslational modification, protein turnover, chaperones’ (6.98%).

The unigenes variance degree between the two genotypes in the same COG categories also exhibited the maximum at the longest treatment time. At 36 HAT, unigenes involved in 18 COG categories in the heat-sensitive genotype had more than two times enrichment than in the heat- tolerant genotype (Figure 2c). At 0 HAT, unigenes just encoding ‘coenzyme transport and metabolism’ in the heat-sensitive tall fescue had more than two times enrichment than in the heat-tolerant tall fescue (Figure 2a). At 12 HAT, unigenes encoding ‘translation, ribosomal structure and biogenesis’ and ‘cytoskeleton’ proteins in the heat-sensitive tall fescue had more than two times enrichment than in the heat-tolerant tall fescue (Figure 2b). Unigenes involved in ‘chromatin structure and dynamics’ and ‘defense mechanisms’ in the heat-tolerant tall fescue had more two times enrichment than in the heat-sensitive tall fescue at 0 HAT and 12 HAT, respectively (Figure 2a, b). Under high-temperature stress, higher enrichment of the tall fescue unigenes in the most of COG categories for heat-sensitive genotypes compared with heat-tolerant ones suggests that thermotolerance was related with the changed expression of genes involved in transcriptional regulation as well as metabolic pathways [4].

### Differential expression of transcripts are affected by high-temperature stress

**Figure 3:**
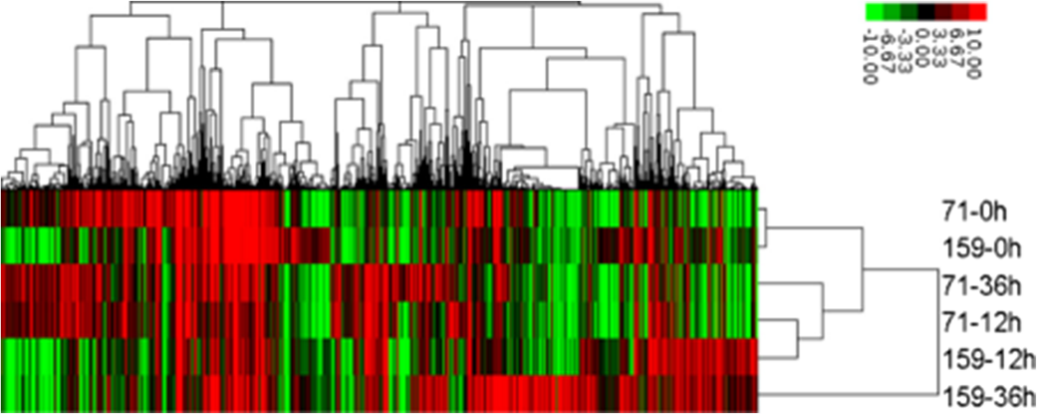
**Average linkage hierarchical clustering analysis of the log2 transformed changed ratio of 31,803 ungenes with Cluster 3.0 software.** The resulting tree figures were displayed using the software package, Java Treeview. Red, up-regulation; green, down-regulation; black, no change. 71–0 h, 12 h and 36 h represent PI 578718 under heat stress for 0, 12 and 36 h; 159–0 h, 12 h and 36 h represent PI 234881 under heat stress for 0, 12 and 36 h.

In order to identify the relationship of genome-wide expression profiles between different genotypes and different treatments, all annotated unigenes from six heat-treated samples were clustered with the software Cluster 3.0 (Figure 3; Additional file 5). The genes detected in the different genotypes or different treatment times were clearly separated. This result further proved that the thermotolerance diversity between PI578718 and PI234881 was regulated by genes, and the sampling at 12 and 36 HAT was suitable to obtain the richest differential expression genes. In addition, heat map of identified genes also showed that the transcript abundance for most of genes under short heat treatment (at 12 HAT) was lower than that under the long heat treatment (at 36 HAT, Figure 3).

**Figure 4:**
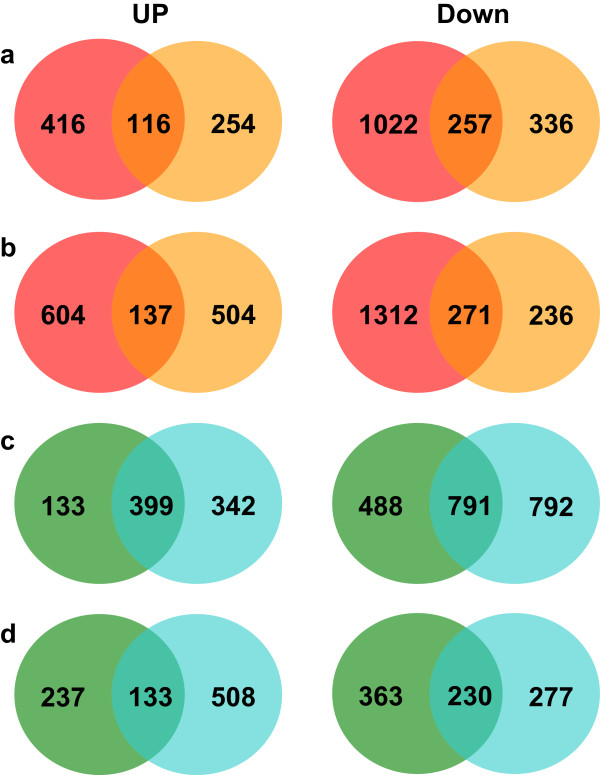
**Venn diagrams showing unigenes up- and down-regulated in tall fescue in response to heat stress.** All these differential genes at heat stress regimes were determined relative to the control regimes (2-fold change, *P* < 0.05, RPKM > 3). **(a)** 12 h, **(b)** 36 h, the left for PI 578718 samples and the right for PI 234881 samples; **(c)** PI 578718, **(d)** PI 234881, 12 h on the left and 36 h on the right.

The Venn diagram analysis of differential expression unigenes in treatment lines relative to the control lines [2-fold change, *P* < 0.05, Reads per kilobase of exon model per million mapped reads (RPKM) > 3] also showed the same result (Figure 4). As shown in Venn diagrams, there were more common genes detected in both genotypes at 36 HAT than at 12 HAT (137 versus 116 for activated; 137 versus 116 for inhibited; Figure 4a, b). For PI 578718, whether up-regulation or down-regulation, the number of differential expression unigenes was larger at 36 HAT than 12 HAT. For PI 234881, the number of activated unigenes was larger at 36 HAT than 12 HAT (Figure 4c, d). The result suggests that the pattern of increased transcript abundance along with the increased stress time was one important response mechanism to high temperature in grass, as previously found for the total of 1,531 genes analyzed by microarray in heat stressed mussels (*Mytilus galloprovincialis*) [22]. In addition, more genes showed decreased transcript abundance regardless of heat tolerant or sensitive genotypes. The result indicates that cool-season turfgrass and forage grass may have more negative regulated genes compared with positive regulation ones in the heat stress response pathway. Qin *et al*. [4] reported that heat-tolerant ‘TAM107’ had more differential expression genes relative to heat-sensitive ‘CS’ (1,011/1,100) at 1 HAT, but less differential expression genes at 24 HAT in wheat using microarray analysis. The results in this study showed that heat-tolerant PI578718 (1,273/2,862) had more differential expression genes relative to heat-sensitive PI234881 (1,011/1,100) at all heat treatment times (Figure 4c, d). The greater genes with differential transcript abundance in the heat tolerant genotype may speak to the complexity of thermotolerance in grass.

### Cell cycle and division are affected by high temperature stress

**Figure 5:**
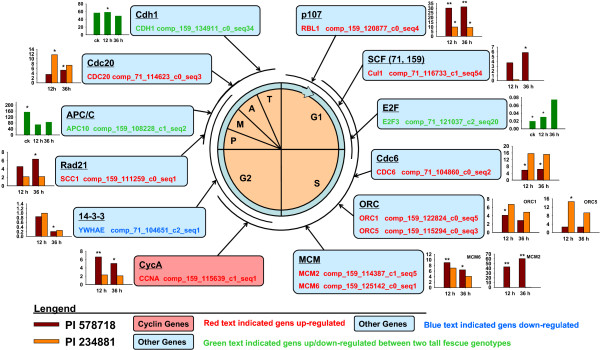
**RNA-Seq expression data in heat-stressed tall fescue associated with cell cycle phases.** Each box represents a gene product, or group of gene products, and indicates where genes in cell cycle affected by heat stress. In the histogram, the expression data for heat-stressed tall fescue present with the fold change value. The green histogram represents the change of gene expression just between two genotypes. Stars above bars indicated significant differences relative to controls.

The expression data annotated by the Kyoto encyclopedia of genes and genomes (KEGG) pathway category “cell cycle” (KO 04110) consisted of 101 unigenes in both tall fescue genotypes in response to high temperature stress (Figure 5). 14 genes of these 101 unigenes showed different transcript abundance. Ten genes were activated in heat tolerant PI578718, but only three genes were activated in heat sensitive PI 234881. Stem cell factor (*SCF*), *p107* and *E2F* have been suggested as the critical regulators of G1 to S-phase progression [23, 24]. The *SCF* gene (*Cul1*, comp_71_116733_c1_seq54) related to elimination of the S-phase cyclin/cyclin-dependent kinase (CDK) inhibitor Sic1 inhibiting cell progress from G1 to S-phase was found to be up-regulated in PI578718 at 36 HAT. Identification of p107 (comp_159_120877_c0_seq4) was shown as one of the critical regulators in the elevation of E2F activity driving cells from G1 into S-phase, and showed increased transcript abundance in two grass genotypes. Although high-temperature stress did not induce significant changes of *E2F3* (comp_71_121037_c2_seq20) expression, more transcript abundance for *E2F3* in PI 578718 was obtained than in PI234881 at 0 and 12 HAT.

In response to heat stress in tall fescue, cell division control protein 6 gene (*CDC6*, comp_71_104860_c0_seq2) initiating DNA replication showed increased expression at 12 and 36 HAT in PI 578718. Under exogenous environment stress, S-phase cells are susceptible to chromosomal damage because of the potential replication errors [25]. Minichromosome maintenance (MCM) and origin recognition complex (ORC) family proteins are essential components in DNA replication licensing and play a key role in preventing centrosome reduplication, particularly for MCM complex in S-phase checkpoint activation and in subsequent lesion repair [25, 26]. At 12 HAT, *ORC1* (comp_159_122824_c0_seq5) and *ORC5* (comp_159_115294_c0_seq3) were activated in PI578718 and PI234881, respectively, but were unaffected in grass leaves at 36 HAT. The transcript abundance of both *MCM2* (comp_159_114387_c1_seq5) and *MCM6* (comp_159_125142_c0_seq1) increased in the heat-stressed PI578718, but were unaffected in heat-stressed PI234881. The result suggests that the heat-tolerant PI578718 had greater ability to self-repair to protect S-phase genome stability than the heat-sensitive PI 234881.

Plant cyclins are typically classified as A, B, C, or D-types [25]. Cyclin A protein is present in the S-phase to M phase and plays a major role in the control of DNA replication, and cyclin D regulates the G1-S transition [27]. Two cyclins [cyclin A (CCNA) and cyclin D3 (CYCD3)] were detected for the first time in tall fescue by genome wide RNA-Seq analysis. *CCNA* (comp_159_115639_c1_seq1) was identified activated in heat-stressed PI578718, unaffected in heat-stressed PI234881. However, *CYCD3* showed decreased transcript abundance in 12 h-treated PI578718. The 14-3-3 proteins are a family of phosphoserine/phosphothreonine binding molecules that promote cell survival and control the function of a wide array of cellular proteins [28]. 14-3-3 epsilon (*YWHAE*, comp_71_104651_c2_seq1) was detected with increased expression at 36 HAT in PI 578718, but not in heat-stressed PI 234881.

One sister-chromatid cohesion gene (*SCC1*) plays a vital role in keeping sister chromatid cohesion at spindle checkpoint until the activation of separase by the anaphase-promoting complex/cyclosome (APC/C) [29]. Cdc20/Fizzy and Cdh1/Fizzy-related complex are critical for the activity of multisubunit E3 ubiquitin ligase APC/C and then triggers the degradation of multiple substrates during mitosis [30]. In this study, *SCC1* (comp_159_122824_c0_seq5) and *CDC20* (comp_71_114623_c0_seq3) showed increased transcript abundance at 36 HAT in PI 578718. *CDC20* showed increased expression at 12 HAT in PI 234881. *APC10* (comp_159_108228_c1_seq2) and E-cadherin gene (*CDH1*) (comp_159_134911_c0_seq34) had more transcript abundance in PI234881 than in PI578718 at 0 and 12 HAT, respectively. However, there was no significant change between PI234881 and PI578718 at 36 HAT. The results suggest that *SCC1*and *CDC20* play more important role in protecting M-phase stability in tall fescue in response to heat stress.

### Energy metabolism is affected by high temperature stress

In the tall fescue leaf, of the 473 annotated unigenes, 164 unigenes in the KEGG category “energy metabolism” were significantly affected by high temperature, mainly involved in six kinds of assimilation pathway: “photosynthesis (KO 00195)”, “photosynthesis-antenna proteins (KO 00196)”, “carbon fixation in photosynthetic organisms (KO 00710)”, “methane metabolism (KO 00680)”, “nitrogen metabolism (KO 00910)” and “sulfur metabolism (KO 00920)”, and one dissimilation “oxidative phosphorylation (KO 00190)” (Additional file 6). Assimilation and dissimilation are the two important aspects of the energy metabolism, and the balance of energy metabolism process between assimilation and dissimilation is fundamental to the maintenance of cell life, the source of its life and development [31, 32]. In “energy metabolism”, most of the genes (75.0%, 66) involved in assimilation, such as “photosynthesis”, “photosynthesis-antenna proteins” and “carbon fixation in photosynthetic organisms” showed decreased transcript abundance in two tall fescue genotypes under heat stress. In the methane, nitrogen and sulfur metabolic pathways, there were also more genes (70.5%, 31) inhibited in the two tall fescue genotypes. However, most of the genes involved in dissimilation, such as “oxidative phosphorylation” showed increased transcript abundance in the heat-tolerant PI 578718 (78.57%, 22) and only inhibited in heat-sensitive PI234881 (66.7%, 6). The result finding indicates that PI578718 obtained more energy than PI234881 in response to high temperature stress. The expression data suggest that greater dissimilation metabolism in PI578718 relative to PI512315 may result in a more stable cell division in PI578718 than in PI234881 in response to high temperature. In the three pathways methane, nitrogen and sulfur metabolism, there were more genes inhibited in PI578718 than in PI234881. One gene encoding cytochrome c oxidase subunit 2 (*COX2*) showed increased abundance in PI 578718, but decreased in PI 234881 under heat stress. The transcript abundance of a ferredoxin gene (*petF*) belonging to this pathway was dramatically increased at all treatment times in PI 578718 but decreased at 36 HAT in PI234881.

### HSP and antioxidant genes are affected by high temperature stress

Plants respond to high temperature by synthesizing a set of the heat shock proteins (HSPs) to acquire thermotolerance [33]. According to their predicted molecular weight, HSPs could be grouped into three families: high molecular weight (HMW-HSP, 80–114 kDa), HSP70 (69–71 kDa), low molecular weight (LMW-HSP, 15–30 kDa) [13]. Many researches have proved that the increased transcript abundance of the three kinds of HSPs and their molecular chaperone was directly involved in the activation of protection mechanisms in response to heat stress [34–37]. However, in contrast with these results, very few *HSP70* genes showed differential expression in the two tall fescue genotypes under heat stress (Additional file 7). Moreover, heat transfer products group (*htpG*) encoding the molecular chaperone HtpG protein and *HSPBP1* encoding hsp70-interacting protein showed decreased transcript abundance at 12 HAT in PI578718. It is interesting to find that nine *HSP20* genes showed increased transcript abundance in all treatment lines for the both genotypes, and two *HSP90B* genes increased abundance in heat-stressed PI 578718, which suggests that the *LMW-HSP* and *HMW-HSP* may play a more important role in enhancing thermotolerance for tall fescue. There is new evidence showing that plants have developed antioxidant enzymatic systems for scavenging antioxidant enzymes to avoid these oxidative injuries induced by abiotic stress [38, 39]. In this study, 14 antioxidant genes, in the KEGG category “peroxisome”, regulating antioxidant enzyme activity, were significantly affected in both heat-stressed genotypes and the tolerant genotype had more antioxidant genes activated than in the sensitive genotype (Additional file 8). Particularly, three *SOD* genes increased transcript abundance at 36 HAT in PI578718 but were unaffected in heat-stressed PI234881. The result suggests that the high expression level of antioxidant genes may be one key functional trait to identify the heat tolerance in tall fescue species.

### Quantitative real-time-PCR validation of differentially expressed transcripts from RNA-Seq

**Figure 6:**
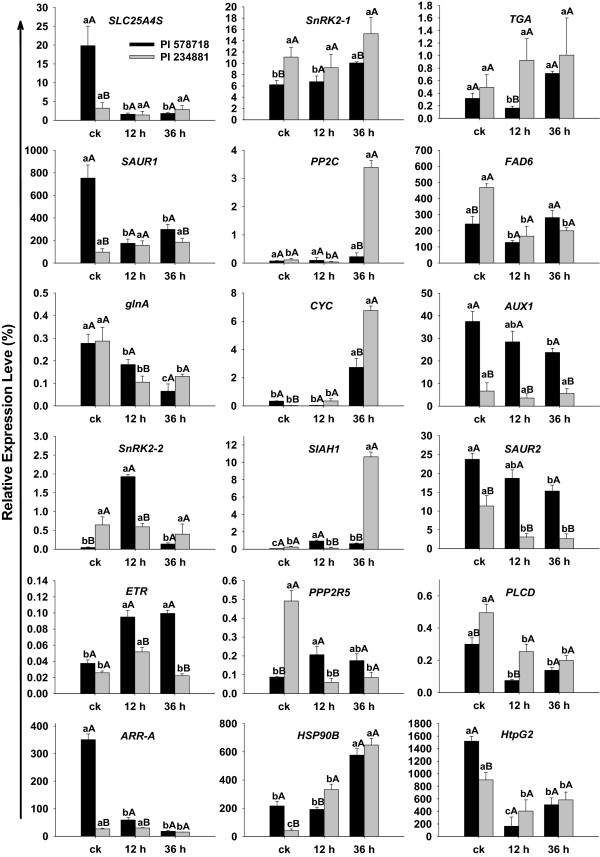
**Expression analysis of 18 randomly selected RNA-Seq genes by qRT-PCR from PI 578718 (deep red bars) and PI 234881 (deep yellow bars).** Green bars represented the different gene expression between two tall fescue genotypes. *YT521-B* gene was used as the reference gene for normalization of gene-expression data. Error bars represent the SE for three independent experiments, and three technical replicates were analysed. Bars with the same lower case letters within a treatment time level indicate are not significant differences; Bars with the same upper case letters within a genotype indicate not significant differences by Fisher’s least significant difference test at *P* < 0.05. CK, 12 h, and 36 h on the x-axis refer to control and 12 and 36 h after heat stress. Gene function and corresponding expression patterns extracted by RNA-Seq are listed in Additional file 6: Table S2.

**Figure 7:**
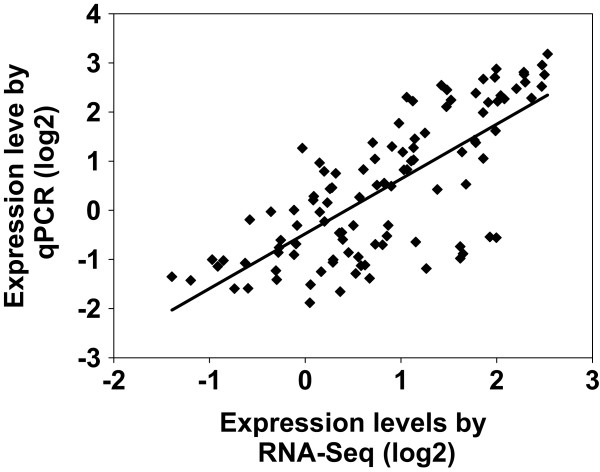
**Correlations of expression level analyzed by log2 RNA-Seq platform (x axis) with data obtained using log2 real-time PCR (qPCR, y axis) for different time points.**

Eighteen randomly selected genes were used to confirm their expression patterns of the Illumina RNA-Seq results by quantitative real-time PCR (qPCR, Figure 6, Additional file 9). Nine genes, encoding SNF-related serine/threonine-protein kinase (*SnRK2-1*, *SnRK2-2*), transcription factor TGA, small auxin upregulated RNA (SAUR) family protein (*SAUR1*, *SAUR2*), protein phosphatase 2C (*PP2C*), auxin influx carrier (*AUX1*), ethylene receptor (ETR) and two-component response regulator ARR-A family (*ARR-A*), were involved in hormone metabolism, while another two genes, encoding 90 kDa beta HSP (*HSP90B*) and molecular chaperone HtpG (*htpG*), were related to HSP metabolism. Two genes encoding protein phosphatase 2 (formerly 2A), regulatory subunit B (*PPP2R5*) and phosphatidylinositol phospholipase C (*PLCD*) were related to protein phosphorylation reaction. The remaining five genes encoded solute carrier family 25 (*SLC25A4S*), omega-6 fatty acid desaturase (*FAD6*), glutamine synthetase (*glnA*), cytochrome c (*CYC*) and E3 ubiquitin-protein ligase (seven in absentia homolog 1, *SIAH1*), respectively. As listed in Additional file 9, eleven unigenes were inhibited and five unigenes were activated in the two grass. The qPCR results showed a similar change trend for all tested unigenes in the heat stressed and non-stressed grass (Figure 6). *SLC25A4S*, *SAUR1* and *AUX1* in PI578718 showed increased transcript abundance in the treatment lines relative to controls. FAD6 in stressed PI 234881 inhibited. Five unigenes (*glnA*, *SAUR2*, *PPP2R5*, *PLCD*, *ARR-A* and *htpG2*) showed decreased transcript abundance in both tall fescue genotypes under heat stress. *CYC*, *SIAH1* and *HSP90B* showed significantly higher expression compared to the controls in two grass genotypes. In PI578718 heat stressed lines, *SnRK2-2* and *SnRK2-1* showed increased transcript abundance at 12 and 36 HAT, respectively. *PP2C* showed increased transcript abundance at 36 HAT in PI234881. *ETR* showed increased transcript abundance at 12 HAT in two grass genotypes. *CYC* and *SIAH1* showed increased abundance at 36 HAT in two grass genotypes. Further, correlation between RNA-Seq and qPCR was evaluated using log2 expression level. As shown in Figure 7, the qPCR measurements were moderately correlated with RNA-Seq results (y = 1.1197x-0.4587; r^2^ = 0.5515; *P* < 0.0001), which indicated that these RNA-Seq data was accuracy and effective, and can be used for gene expression profiles analysis during the tall fescue high temperature defence response.

## Conclusions

In conclusion, this study provided the first large-scale transcriptome dataset in cool-season turfgrass in response to high-temperature stress. More than 262 million high-quality 100-bp paired-end reads paired-end reads were generated and assembled 31,803 unigenes in tall fescue. Furthermore, 12,974 unigenes expressed specifically in tall fescue under high-temperature stress, as well as their classification, functions, and metabolic pathways were shown for the first time. High expression levels of genes involved in LMW (HMW)-HSP, antioxidants, cell cycle and cell division and dissimilation (oxidative phosphorylation) in tall fescue respond to heat stress could play a vital role in achieving thermotolerance.

## Methods

### Plant materials and growth conditions

Healthy and single clonal plants of two tall fescue (*Festuca arundinacea* Schreb.) genotypes, heat-tolerant PI578718 and heat-sensitive PI234881 were collected from turfgrass field plots at Wuhan Botanical Garden, Chinese Academy of Sciences, Wu Han, Hubei, China. Tall fescue plants were grown in plastic pots (13 cm diameter, 11 cm deep) with a mix of sand and peat soil (1/1, v/v) in the greenhouse under natural sunlight, relative humidity of 87%, wind speed of 0.8 m · s^−1^ and temperatures of 22/18°C (day/night). After 3 months of establishment, plants were transferred into controlled-environment growth chambers (HP300GS-C; Ruihua Instrument, Wuhan, China), with a 14-h photoperiod, photosynthetically active radiation at 450 μmol m^−2^ s^−1^ at the canopy level, a day/night temperature of 22/18°C and 70% relative humidity. The plants were allowed to acclimate for one week before they were exposed to heat treatments. Throughout the experiment, the plants were fertilized three times a week with half-strength Hoagland’s solution [40] until dripping occurred.

### Treatments and experimental design

The plant-pot system was weighed at 48-h intervals to determine transpiration rate (T_r_) before heat treatment initiation based on the method described by Hu *et al*. [39]. All plants from each tall fescue genotype were divided into two groups with similar T_r_ (Group I, II). Group I of tall fescues was kept at 22/18°C day/night in the controlled-environment growth chambers. Group II was subjected to heat treatments at 40°C in the controlled-environment growth chambers (except for temperature, the other conditions remained the same as the control). At the beginning of heat treatment, all plots were hand irrigated until dripping occurred; upon reaching this point, the irrigation was withheld during the experiment. Heat treatment began at 7: 00 am and it took about 45 min for the temperature to increase from 22°C to 40°C. The plants were exposed to prolonged 40°C heat stress for 36 h at 450 μmol m^−2^ s^−1^ and 70% relative humidity. The leaf samples for Illumina deep sequencing analysis were collected at 12 and 36 h after plants were exposed to 40°C, respectively. At each sampling time point, the leaf tissues from the three pots (three replicates, one pot per replicate) of each genotype pooled together as one biological replicate and froze immediately with liquid nitrogen, and then stored at −80°C for RNA-Seq analysis. A total of 12 control samples including the leaves collected at 0 h and 22°C in Group II were pooled together as the sample at 0 h for each genotype. A total of six samples in total were used for Illumina Genome Analyzer deep sequencing and 6 G data performed for each sample.

### Total RNA, Poly(A) RNA Isolation, and Library Preparation

Total RNA from tall fescue leaf tissues was extracted separately using Trizol reagent (Invitrogen, Carlsbad, CA) and purified with the RNeasy Plant Mini Kit (Qiagen, Valencia, CA). The concentration and quality of the total RNA was determined by a NanoDrop 8000 spectrophotometer (NanoDrop, Wilmington, DE) and checked by running a gel electrophoresis in 1.5% (w/v) agarose gels. According to the manufacturer’s instructions (Illumina, San Diego, CA), the poly (A) mRNA was isolated from purified total RNA using biotin-Oligo (dT) magnetic beads and fragmented into small pieces using an RNA fragmentation kit. First-strand cDNA was generated from the cleaved RNA fragments using reverse transcriptase and random primers. Second-strand cDNA was synthesized using RNase H and DNA Polymerase I. Following adaptor ligation, the unsuitable fragments were removed with AMPureXP beads, 200-bp cDNA fragments were enriched by 18 cycles of PCR and checked with Pico green staining quantified with Agilent 2100 Bioanalyzer (Agilent Technologies, Palo Alto, CA). The products were sequenced on the Illumina HiSeq 2000 instrument in Shanghai Personal Biotechnology Co., Ltd.

### Processing and Mapping of Illumina Reads

The RNA-Seq raw reads were processed to obtain high-quality reads by removing the adapter sequences and low-quality bases at the 3’ end, trimming low-quality bases (Q < 20) from the 5’ and 3’ ends of the remaining reads. Reads filtering out reads containing ‘N’ and greater than 25 bp were considered for analysis. Clean reads were assembled into contigs, transcripts and unigenes with Trinity software (http://trinityrnaseq.sf.net). RPKM was used to normalize the abundances of transcripts [41]. More than a 2-fold change was used to identify the significance of different gene expression between different treatment lines.

### Identification of differentially expressed unigenes

All unigenes were blastx searched against Brachypodium hereafter (*Brachypodium distachyon*), wheat (*Triticum* spp.), rice (*Oryza sativa* Linn.), maize (*Zea mays* L.), sorghum [*Sorghum bicolor* (L.) Moench] in KEGG and eggNOG protein databases (E-value < 10^−5^) and functionally annotated by Blast2GO Gene Ontology Functional Annotation Suit (E-value < 10^−5^) (http://www.blast2go.org/). Metabolic pathways were predicted by KEGG mapping. Putative transcription factors were identified by searching *Arabidopsis* Gene Regulatory Information Server (AGRIS) Database [42].

### Cluster analysis

Hierarchical clustering analysis was carried out for all annotated transcripts from heat tolerant PI578718 and heat sensitive PI234881. The RPKM counts for each transcript were clustered with the software Cluster 3.0, and JAVA Treeview was used to view the cluster image. The results were visualized using JAVA Treeview [43].

### Validation of RNA-Seq by qRT-PCR

First-strand cDNA was generated from 3 μg of the same total RNA used by RNA-Seq analysis. The expression level of the target genes was determined by means of quantitative Real-Time PCR (qPCR) using ABI StepOne Plus Real-Time PCR system (Applied Biosystems, Foster City, CA) and SYBR Green Real-Time PCR Master Mix (Toyobo, Osaka, Japan) in 20 μL reactions. Each reaction consisted of a 2 ng of total RNA, 0.5 μL of each primer and 10 μL master mix. The PCRs were performed in thermocycler conditions starting with 3 min at 95°C, 40 cycles of 10 s at 94°C, 20 s at 55°C, and 20 s at 72°C, followed by 5 min at 72°C. To verify the presence of a specific product, the melting curve analysis of amplification products was performed at the end of each PCR reaction. In addition, the size of each amplified DNA fragment was verified on a 1.5% (w/v) agarose–ethidium bromide gel at 100 V for 40 min in 1 × TE buffer (10 mM Tris, 1 mM EDTA). Primers (see Additional file 8) for qPCR were designed with Primer Premier software (Primer Premier v5.0; Premier Biosoft International, Palo Alto, Calif.). The comparative CT method of quantification was used to quantify the relative expression of specific genes [44]. *YT521-B* gene was used as references [45]. The experiments were repeated twice with three replicates.

To assess the correlation between different platforms, the Pearson correlations were calculated by SPSS 16.0 to compare the mRNA expression levels measured by RNA-Seq and qPCR. To assess treatment effects between different treatment lines, the variance analysis was performed using the Statistical Analysis System (SAS 9.0 for windows, SAS Institute Inc., Cary, NC). The mean separation was performed with Fisher’s least significant difference test at *P* < 0.05.

## Electronic supplementary material

Additional file 1: **Analysis of physiological acclimation in leaves of two tall fescue genotypes (heat-tolerant PI 578718 and heat-sensitive PI 234881) affected by heat stress.** (a) Phenotypes of two tall fescue genotypes exposed to 40°C at 36 h after treatment (HAT). (b) Phenotypes of two tall fescue genotypes exposed to 38/30°C (day/night) at 14 days after treatment (DAT). (c) Analysis of physiological traits in leaves of two tall fescue genotypes exposed to 40°C at 36 HAT. Means in a column followed by the same lower-case letter for each treatment line are not significant; means in a row followed the same upper-case letters for each genotype are not significant at Fisher’s least significant difference test at *P* < 0.05. (DOC 1 MB)

Additional file 2: **Functional categorization of entire assembled unigenes based on Gene Ontology (GO) classification in all treatment lines of two genotypes (heat-tolerant PI 578718 and heat-sensitive PI 234881).** The unigenes were summarized in three main GO categories (biological process, cellular component and molecular function) and 54 subcategories. The y-axis indicates the numbers of unigenes in each class and the x-axis indicates the subcategories. (DOC 166 KB)

Additional file 3: **Functional categorization of assembled unigenes based on Gene Ontology (GO) classification for 0 h heat-stressed PI 578718 and PI 234881.** The unigenes were summarized in three main GO categories (biological process, cellular component and molecular function) and 54 subcategories. The y-axis indicates the numbers of unigenes in each class and the x-axis indicates the subcategories. (DOC 166 KB)

Additional file 4: **Functional categorization of assembled unigenes based on Gene Ontology (GO) classification for 12 h heat-stressed PI 578718 and PI 234881.** The unigenes were summarized in three main GO categories (biological process, cellular component and molecular function) and 54 subcategories. The y-axis indicates the numbers of unigenes in each class and the x-axis indicates the subcategories. (DOC 174 KB)

Additional file 5: **Gene expression analysis of differential expression genes used for linkage hierarchical clustering analysis.** (XLS 1017 KB)

Additional file 6: **Gene annotation and expression analysis in Energy Metabolism which includes Oxidative phosphorylation, Photosynthesis, Photosynthesis-antenna proteins, Carbon fixation in photosynthetic organisms, Methane metabolism, Nitrogen metabolism, Sulfur metabolism.** (XLS 56 KB)

Additional file 7: **Gene annotation and expression analysis in the heat shock protein metabolism.** (XLS 23 KB)

Additional file 8: **Gene annotation and expression analysis in the peroxisome metabolism.** (XLS 20 KB)

Additional file 9: **Gene information used for qRT-PCR verification of RNAseq data and corresponding expression patterns extracted by RNA-Seq.** (XLS 26 KB)
