# Supplementary material for: An RNA sequencing transcriptome analysis of the high-temperature stressed tall fescue reveals novel insights into plant thermotolerance
Source: BMC Genomics. 2014 Dec 19;15(1):1147. doi: 10.1186/1471-2164-15-1147 (PMC4378353; doi:10.1186/1471-2164-15-1147)
Supplement: Supplementary file 1 — Additional file 1: Analysis of physiological acclimation in leaves of two tall fescue genotypes (heat-tolerant PI 578718 and heat-sensitive PI 234881) affected by heat stress. (a) Phenotypes of two tall fescue genotypes exposed to 40°C at 36 h after treatment (HAT). (b) Phenotypes of two tall fescue genotypes exposed to 38/30°C (day/night) at 14 days after treatment (DAT). (c) Analysis of physiological traits in leaves of two tall fescue genotypes exposed to 40°C at 36 HAT. Means in a column followed by the same lower-case letter for each treatment line are not significant; means in a row followed the same upper-case letters for each genotype are not significant at Fisher’s least significant difference test at P < 0.05. (DOC 1 MB) [file 12864_2014_6885_MOESM1_ESM.doc]

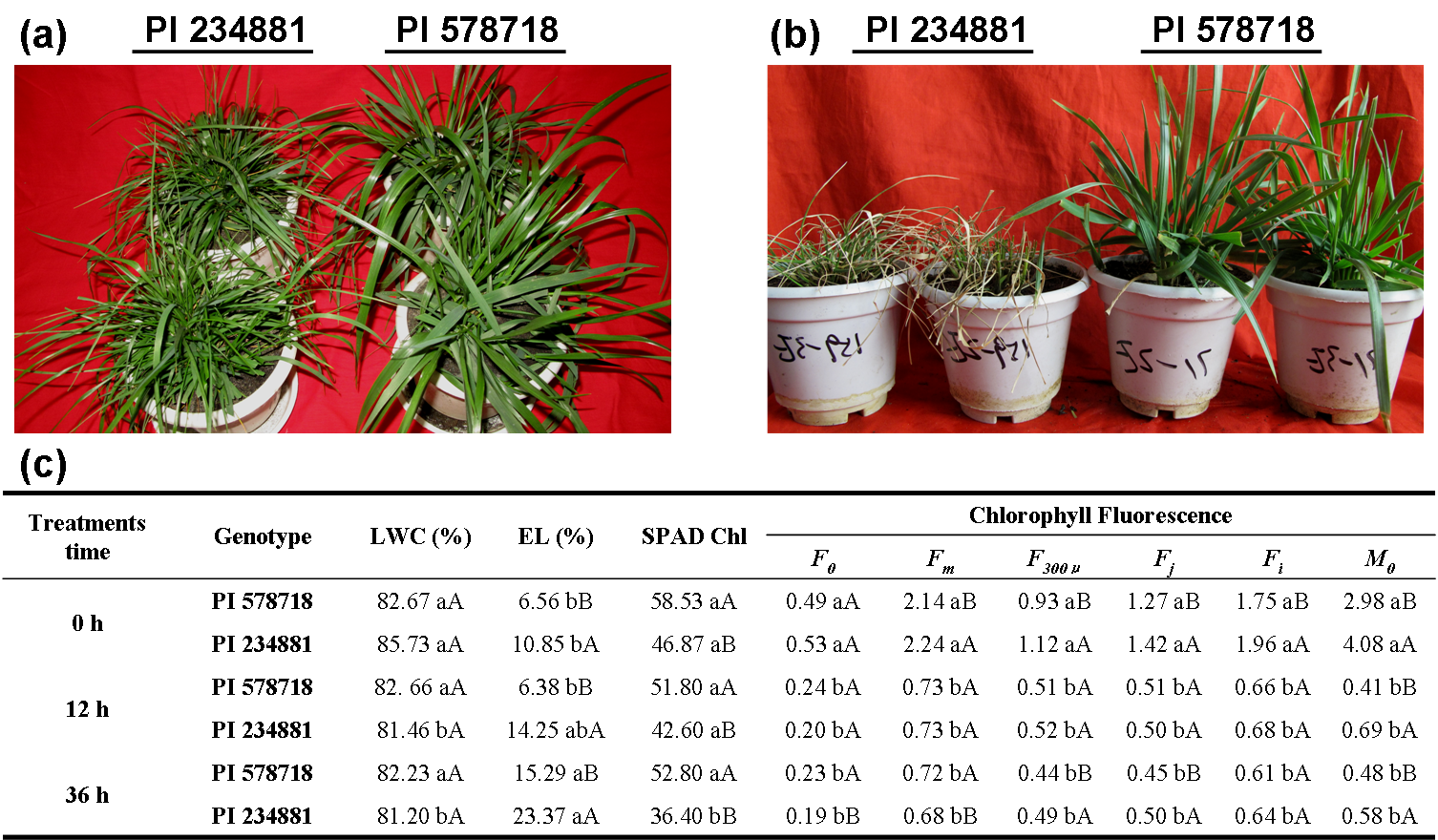


**Additional file 1** Analysis of physiological acclimation in leaves of two tall fescue genotypes (heat-tolerant PI 578718 and heat-sensitive PI 234881) affected by heat stress. (a) Phenotypes of two tall fescue genotypes exposed to 40 °C at 36 h after treatment (HAT). (b) Phenotypes of two tall fescue genotypes exposed to 38/30 °C (day/ night) at 14 days after treatment (DAT). (c) Analysis of physiological traits in leaves of two tall fescue genotypes exposed to 40 °C at 36 HAT. Means in a column followed by the same lower-case letter for each treatment line are not significant; means in a row followed the same upper-case letters for each genotype are not significant at Fisher’s least significant difference test at *P* < 0.05.
