# Supplementary material for: An RNA sequencing transcriptome analysis of the high-temperature stressed tall fescue reveals novel insights into plant thermotolerance
Source: BMC Genomics. 2014 Dec 19;15(1):1147. doi: 10.1186/1471-2164-15-1147 (PMC4378353; doi:10.1186/1471-2164-15-1147)
Supplement: Supplementary file 4 — Additional file 4: Functional categorization of assembled unigenes based on Gene Ontology (GO) classification for 12 h heat-stressed PI 578718 and PI 234881. The unigenes were summarized in three main GO categories (biological process, cellular component and molecular function) and 54 subcategories. The y-axis indicates the numbers of unigenes in each class and the x-axis indicates the subcategories. (DOC 174 KB) [file 12864_2014_6885_MOESM4_ESM.doc]

**Additional file 4** Functional categorization of assembled unigenes based on Gene Ontology (GO) classification for 12 h heat-stressed PI 578718 and PI 234881. The unigenes were summarized in three main GO categories (biological process, cellular component and molecular function) and 54 subcategories. The y-axis indicates the numbers of unigenes in each class and the x-axis indicates the subcategories.
